# The Added Value of Operator's Judgement in Thyroid Nodule Ultrasound Classification Arising From Histologically Based Comparison of Different Risk Stratification Systems

**DOI:** 10.3389/fendo.2020.00434

**Published:** 2020-07-07

**Authors:** Bruno Madeo, Giulia Brigante, Anna Ansaloni, Erica Taliani, Shaniko Kaleci, Maria Laura Monzani, Manuela Simoni, Vincenzo Rochira

**Affiliations:** ^1^Unit of Endocrinology, Department of Biomedical, Metabolic and Neural Sciences, University of Modena and Reggio Emilia, Modena, Italy; ^2^Department of Medical Specialties, Azienda Ospedaliero-Universitaria di Modena, Modena, Italy; ^3^Department of Diagnostic Medicine, Clinics and Public Health, Azienda Ospedaliero-Universitaria di Modena, Modena, Italy

**Keywords:** ultrasound, thyroid nodules, classifications, malignancy risk, histology

## Abstract

**Objective:** Several ultrasound classifications for thyroid nodules were proposed but their accuracy is still debated, since mainly estimated on cytology and not on histology. The aim of this study was to test the diagnostic accuracy and the inter-classification agreement of AACE/ACE-AME, American Thyroid Association (ATA), British Thyroid Association (BTA), and Modena Ultrasound Thyroid Classification (MUT) that stratifies malignancy risk considering also the clinician subjective impression.

**Methods:** A prospective study collecting thyroid nodule features at ultrasound and histological diagnosis was conducted. Ultrasound features were collected following a preformed checklist in candidates for surgery because of indeterminate, suspicious, or malignant cytology. All the nodules, besides the cytologically suspicious one, were blinded analyzed. MUT score was applied prospectively, and the others retrospectively. Sensitivity, specificity, diagnostic cut-off value, and accuracy of each classification were calculated. The overall agreement between classifications was tested by Bland-Altman, and agreement between single nodule analysis by different classifications by Weighted Cohen's Kappa.

**Results:** In classifying a total of 457 nodules, MUT has the highest accuracy (AUC 0.808) and specificity (89%), followed by ATA and BTA, and finally by AACE/ACE-AME. ATA, BTA, and MUT are highly interchangeable. Considering agreement between single nodule analyses, ATA and BTA had the best (κ = 0.723); AACE/ACE-AME showed slight agreement with BTA (κ = 0.177) and MUT (κ = 0.183), and fair agreement with ATA (κ = 0.282); MUT had fair agreement with both ATA (κ = 0.291) and BTA (κ = 0.271).

**Conclusion:** Classifications have an acceptable overall diagnostic accuracy, improved using a less rigid system that takes into consideration operator subjective impression.

## Introduction

The leading role of ultrasound (US) in thyroid nodules evaluation is now well stated and accepted. However, no single US feature was proven to be unequivocally predictive of benignity or malignancy and to provide reliable information to categorically select nodules that should undergo fine needle aspiration (FNA) (1). Consequently, only the combination of different US characteristics can identify nodules with an increased risk for malignancy.

Recent meta-analyses demonstrated that clinical studies failed to identify US features that, alone or in combination, are certainly indicative of malignancy or benignity (1–3). The highest odds ratio for malignancy was found for taller than wide shape, microcalcifications, irregular margins, and absence of elasticity or halo sign. On the other hand, only cystic content and spongiform appearance seem to predict benign nodules. A remarkable heterogeneity across studies was detected, increasing the likelihood of bias and reducing the reliability of the estimated diagnostic accuracy.

Several US classifications have been proposed with the aim to provide a useful tool for both clinicians and researchers (4–17). More recently, the AACE/ACE-AME Task Force on thyroid nodules (15) reviewed and compared classifications adopted by the American Thyroid Association (ATA) in 2015 (16) and by the British Thyroid Association (BTA) in 2014 (17), offering a new proposal weighted on current scientific evidence. Eventually, a three-class rating system was proposed, distinguishing low, intermediate, and high-risk lesions (15). Moreover, they suggest to complete US reports with a rating that stratifies nodules based on their malignancy risk (15).

In order to have an orientation in this maze of classifications, some studies recently compared their predictive value of malignancy (18–21). However, results are not univocal, and accuracy has almost always been estimated considering nodule cytology and only rarely, moreover retrospectively, final histological diagnosis (22). Moreover, clinical practice and some studies suggested that most of these systems are not always easily applicable, due to their intrinsic low flexibility. For example, up to 5% of nodules do not match any sonographic pattern proposed by ATA and remain in a gray area (14, 18, 19).

Before the risk stratification systems mentioned above were published, we set up a local classification, based on the existing literature on the predictive US characteristics of malignancy. This tool named Modena US Thyroid Classification (MUT) considers, in the last instance, also the subjective impression of the clinician for differentiating low-risk from high-risk thyroid nodules, based on his/her own clinical experience with the aim to add information especially for those nodules with uncertain categorization due to US characteristics within the above-mentioned gray area.

The aim of the present study was to test the diagnostic accuracy of different thyroid US classification systems (ATA, AACE/ACE-AME, BTA, and MUT) and to evaluate inter-classification agreement by using histological outcomes as term of comparison. In particular, the effect of the operator judgment in the MUT classification and the histological outcome represent novelty with respect to previous studies comparing different US classifications of thyroid nodules.

## Methods

### Study Design

From November 2008 to April 2015, we prospectively enrolled patients who underwent US-guided FNA with a cytological diagnosis of indeterminate (TIR3), suspicious for malignancy (TIR4), or malignant (TIR5) lesion, according to the Italian Consensus for the cytological classification of thyroid nodules (23), on one or more thyroid nodules. The samples collected before the use of this classification have been revised and a specific cytological category was assigned. Each cytological sample was analyzed by expert physicians, in the same Pathology laboratory.

We selected 111 patients with the following inclusion criteria: age above 18 years; indication for thyroid surgery accordingly to a cytological diagnosis of TIR3 or TIR4 or TIR5 on at least one thyroid nodule.

**Figure 1:**
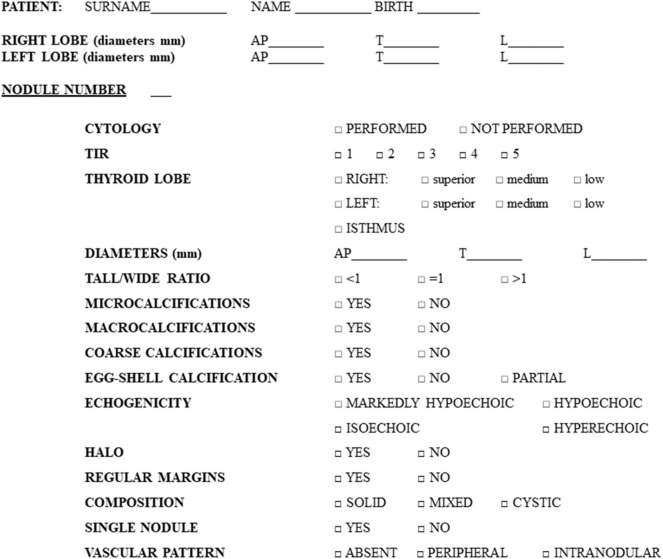
Preformed checklist used for the collection of nodules features during ultrasound examination.

All the enrolled patients underwent neck US before surgery with the purpose of carefully describing each nodule and explore neck region lymph nodes. US examination was performed using a checklist (Figure 1) for collecting each US feature in detail (see section US examination). When multinodular goiter was present, each nodule, in addition to the one subjected to FNA, was carefully described, following the listed items, and mapped in relation to its position within thyroid making the comparison with histology possible. In particular, the precise position of each nodule in the lobes or isthmus was recorded. During the exam, the endocrinologist classified each nodule on the basis of US features and his own impression in five categories (MUT 1–5), detailed below (see section US classifications). After US, all the enrolled patients underwent total thyroidectomy, with or without lymph nodes dissection based on clinical indication. For each nodule, we therefore obtained a histological confirmation and exhaustive description of its location. Thus, the histological report was examined to match the US description with the final diagnosis for each nodule, thanks to the detailed information about nodules position in both reports.

Finally, we retrospectively classified all the US scanned nodules according to AACE/ACE-AME, ATA, and BTA classification systems.

The study was approved by the local ethical committee “Comitato etico provinciale di Modena,” and all participants signed an informed consent.

### US Examination

US scans were performed with Siemens Acuson Antares® (Philadelphia, USA, 10 MegaHertz-linear scanner, B mode) by a single expert endocrinologist, with 10 years of thyroid US experience at study baseline, aware of the presence of a suspect nodule. In each patient, nodules were described, regardless of their cytological diagnosis, for a total of 457 lesions (see section Results for details) and specific features were collected in a preformed checklist (Figure 1). In order to reduce errors, all the data collected were recorded in real time, reducing the risk of forgetting the analysis of some US feature. In other words, the checklist obliged the operator to check all the itemized US-features for each nodule (Figure 1).

The following US features were considered: nodule localization (position in the right lobe/left lobe/isthmus), size, shape, calcifications, echogenicity, margins, composition, vascular pattern, and the presence of uninodular or multinodular goiter. In particular, size was described with 3 diameters: antero–posterior, transverse, and longitudinal (reported in mm). Shape was considered as a tall/wide ratio >1 or <1. The presence or absence of calcifications was described as follows: microcalcifications (<2 mm), macrocalcifications (>2 mm), eggshell calcification, coarse calcifications. The ultrasound echogenicity was defined compared to the surrounding parenchyma as: hyperechoic, isoechoic, hypoechoic, or markedly hypoechoic (compared to muscle echogenicity). Margins were described as regular or irregular (including microlobulated, speculated, and infiltrative). The presence or the absence of a peripheral hypoechoic halo was specified. Considering composition, we described nodules as solid, cystic, or mixed. Vascularization was measured by color-Doppler and indicated as absent, peripheral, or intranodular.

### US Classifications

During US scan, performed after the suspect cytological result and before surgery, the endocrinologist classified each nodule according to MUT classes: MUT1, unclear nodular lesion (e.g., pseudonodular appearance in Hashimoto thyroiditis); MUT2, nodule without features suspicious for malignancy (cystic or spongiform appearance); MUT3, indeterminate nodule (nodular lesion not attributable to MUT2, MUT4, or MUT5 categories); MUT4, suspect nodule (with at least one of microcalcifications, irregular margins, hypoechogenicity, intranodular vascularization, tall/wide ratio > 1, incomplete eggshell calcification with extension to soft tissue, clear extrathyroid extension); MUT5, very suspect nodule (with one or more of the features listed for MUT4, and considered strongly suspect according to operator's judgment). These categories were defined using the structure of the Italian Consensus for the thyroid nodules cytological classification (23) as a model, in order to make its use and interpretation easier. Specifications of each of the five categories were defined according to the knowledge present in the literature, leaving the US operator the possibility to rule on the nodule category risk based on his ultrasound-clinical experience. Starting from the concept that there is inter-observer and intra-observer variability in interpreting US characteristics and that available US classifications do not cover all US characteristics (24, 25), the operator was asked to downgrade or upgrade the class of risk in presence of its judgment based also on clinical (not US) data available and on his advisory opinion arising from his/her US series of experience.

In addition, thanks to the detailed collection of each nodule US features on the preformed checklist, we were able to assign a category risk according to AACE/ACE-AME, ATA, and BTA US classification systems (15–17).

In summary, AACE/ACE-AME classification includes the following risk classes: low-risk (AACE/ACE-AME 1), intermediate-risk (AACE/ACE-AME 2), and high-risk (AACE/ACE-AME 3) lesions (15). The ATA classification discriminates among the following categories: benign, purely cystic nodules (ATA1); very low suspicion nodules without any of the US features described in low-, intermediate-, or high-suspicion patterns (ATA2); low suspicion (ATA3); intermediate suspicion (ATA4); high suspicion (ATA5) (16). The BTA classification recommends the following five categories: normal thyroid tissue (BTA1); benign nodules (BTA2); indeterminate/equivocal nodules (BTA3); suspicious nodules (BTA4); malignant nodules (BTA5) (17).

### Statistical Analysis

The Cohen's kappa (κ) statistic was used to measure the agreement between measurements of the classification systems. We selected κ statistic as the measure of agreement because our variable of interest is categorical (26, 27). Kappa is a measure of this difference, standardized to lie on a −1 to 1 scale, where 1 is perfect agreement, 0 is exactly what would be expected by chance, and negative values indicate agreement less than chance, i.e., potential systematic disagreement between the observers. The interpretation of agreement adopted here is less than chance agreement (κ = 0), slight agreement (κ = 0.01–0.20), fair agreement (κ = 0.21–0.40), moderate agreement (κ = 0.41–0.60), substantial agreement (κ = 0.61–0.80), and almost perfect agreement (κ = 0.81–0.99).

The diagnostic performance is evaluated on the receiver operating characteristic (ROC) curve and the area under the curve (AUC). A ROC curve describes the relationship between the sensitivity and specificity of a test by plotting the two against one another while varying the evaluation, which determines the outcome of a test. The two are inversely related, as one increases the other decreases. Conventionally, since both values range between 0 and 1, the sensitivity (true positive rate) is plotted against 1 minus the specificity (false positive rate). The plot is, therefore, in essence, a representation of the tradeoff between detecting true and false positive cases. Cut-off was calculated for each classification and then used to compare the rankings with the histological diagnosis. Moreover, Bland-Altman analyses were used to validate agreement between the four measurements of the classification systems. The Bland–Altman scatter plot represents the relationship between the values of the differences of two measurements of the same nature (*y*-axis) and their mean (*x*-axis), indicating the line relative to the average of the differences of the two measurements ± 2 standard deviation.

For all analyses, a *p* < 0.001 was considered statistically significant. MedCalc Statistical Software version 14.8.1 (MedCalc Software bvba, Ostend, Belgium; http://www.medcalc.org; 2014) and STATA program version 14 (StataCorp LP 4905 Lakeway Drive College Station, Texas 77845, USA) were used to perform statistical analysis.

## Results

A total of 111 patients (33 males, 78 females; mean age 51 years) were enrolled in the study. Fifteen subjects had uninodular goiter and 96 were affected by multinodular goiter. In the latter, all 442 nodules were US evaluated and classified. Considering solitary and multiple nodules together, a total of 457 nodules were analyzed. Cytological characteristics and malignancy rate at histology are summarized in Table 1.

**Table 1:** Characteristics of the analyzed thyroid nodules.

	**Solitary nodules**	**Nodules in**
	**(*n* = 15)**	**multinodular goiter**
		**(*n* = 442)**
Analyzed by FNA	15/15 (100%)	156/442 (35%)
Cytology^*^		
TIR1	0	21 (13%)
TIR2	0	29 (19%)
TIR3	3 (20%)	40 (26%)
TIR4	5 (33%)	42 (27%)
TIR5	7 (47%)	24 (15%)
**Histology**		
Malignancy rate	14/15 (93%)	103/156 (66%)
Not analyzed by FNA	0	286/442 (65%)
**Histology**		
Malignancy rate	n.a.	17/286 (6%)

*Cytological classification was available only for nodules that underwent fine needle aspiration (FNA). Malignancy rate was calculated considering histological diagnosis for each nodule, after surgery*.

* *According to “Italian Consensus for the cytological classification of thyroid nodules” (23): TIR1, non-diagnostic; TIR2, negative for malignant cells; TIR3, inconclusive/indeterminate; TIR4, suspicious for malignancy; TIR5, diagnostic of malignancy. n.a., not applicable*.

Overall, histological examination revealed 323 benign nodules (71%) and 134 malignant nodules (29%), comprising 80 classic papillary carcinomas, 4 cystic papillary carcinomas, 29 follicular variants of papillary carcinoma, 5 oncocytic papillary carcinomas, 6 follicular carcinomas, 2 Hurthle cells carcinomas, 1 insular carcinoma, and 7 medullary carcinomas.

**Figure 2:**
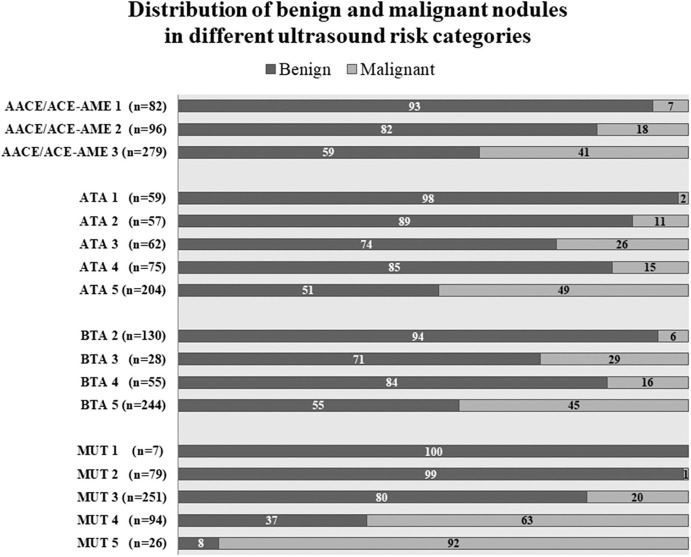
Nodules distribution according to AACE/ACE-AME Task Force on thyroid nodules, American Thyroid Association (ATA), British Thyroid Association (BTA), and Modena US Thyroid Classification (MUT). Benignity and malignancy have been diagnosed histologically according to histology. Numbers in bars are expressed as percentage of the total number of nodules for each category.

Thanks to a detailed description of nodule position, it was possible to establish the correspondence between sonographic appearance, cytology, and histology for each lesion. Nodules distribution according to the four studied classification systems is shown in Figure 2, together with the percentage of benign or malignant lesions for each category. As expected, malignancy rate increases in each category with increasing risk class, with only one deviation in category 4 of ATA and BTA. In this case, malignancy rate is reduced compared to previous category, suggesting a reduction in specificity, confirmed below in our subsequent analyses. Considering the highest risk class for each classification, malignancy rate was between 41 and 50% in ATA, AACE/ACE-AME, and BTA, and highest (92%) only in MUT.

Among the 134 malignant nodules, 34 were in the context of a multifocal neoplasia. Only 78 were expected from cytology (TIR4 or TIR5), while 56 were unexpectedly diagnosed.

**Figure 3:**
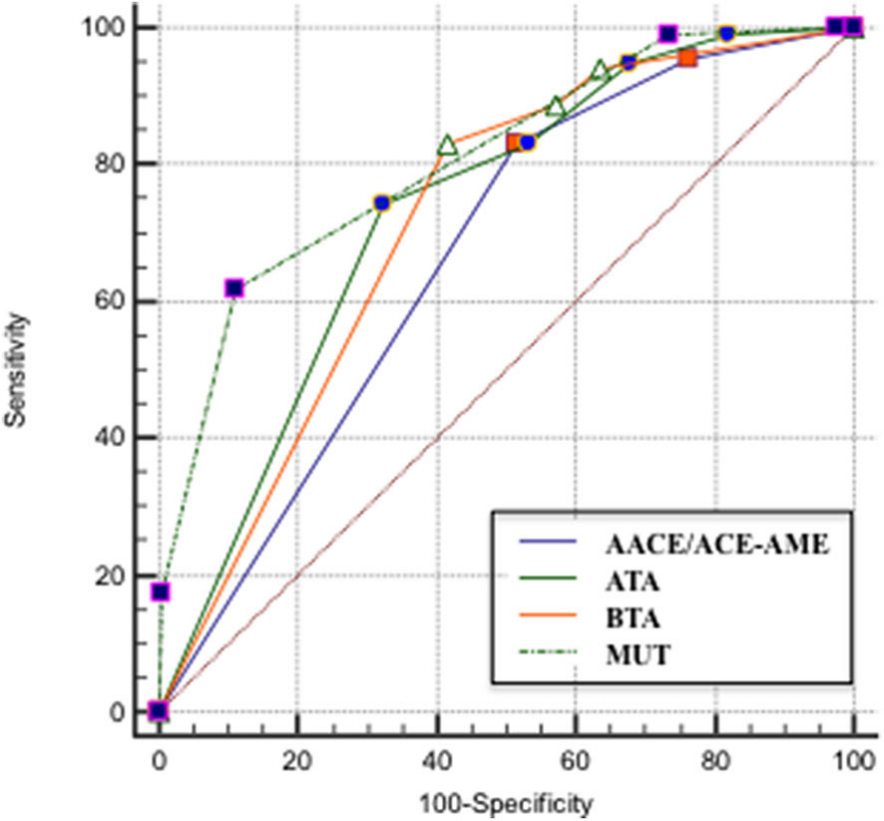
ROC curve that describes the relationship between the sensitivity and specificity of the four classification systems [AACE/ACE-AME Task Force on thyroid nodules, American Thyroid Association (ATA), British Thyroid Association (BTA), and Modena US Thyroid Classification (MUT)].

The ROC curve analysis demonstrated that MUT classification system has the highest AUC, followed by ATA, BTA, and finally, AACE/ACE-AME (Figure 3). Sensitivity, specificity, AUC, and the cut-off to better predict the risk of malignancy for each classification are shown in Table 2. In particular, the analysis indicated that categories above two for AACE/ACE-AME, above three for MUT, and above four for ATA and BTA are the best to predict malignancy risk, having the best combination of sensitivity and specificity.

**Table 2:** ROC curve analysis for the different classification systems.

	**Sensitivity**	**Specificity**	AUC^*^	**Cut-off**
AACE/ACE-AME	83	48	0.666	>2
ATA	74	68	0.731	>4
BTA	83	58	0.718	>4
MUT	62	89	0.808	>3

*AACE/ACE-AME Task Force on thyroid nodules, American Thyroid Association (ATA), British Thyroid Association (BTA), and Modena US Thyroid Classification (MUT)*,

* *AUC, area under the curve*.

**Figure 4:**
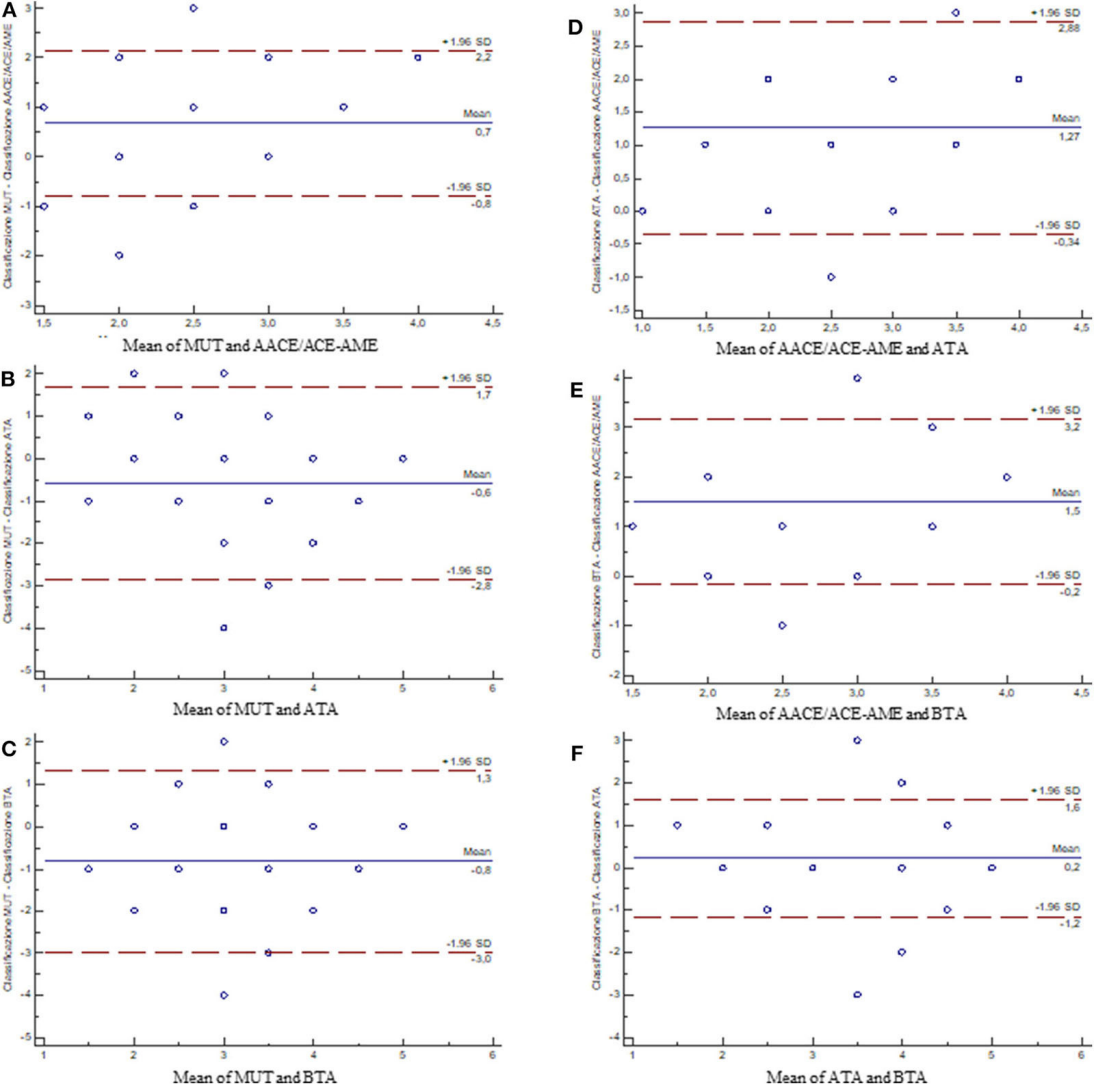
Bland–Altman plots showing the differences between measurements of the classification systems. The blue line is the average of the differences (in case the first and second measurements were coincidentally, points would be aligned along the axis of the abscissas and positioned on the value 0); the dot lines are the 95% limits of agreement. **(A)** MUT vs. AACE/ACE-AME; **(B)** MUT vs. ATA; **(C)** MUT vs. BTA; **(D)** AACE/ACE-AME vs. ATA; **(E)** AACE/ACE-AME vs. BTA; **(F)** ATA vs. BTA [AACE/ACE-AME Task Force on thyroid nodules, American Thyroid Association (ATA), British Thyroid Association (BTA), and Modena US Thyroid Classification (MUT)].

The quantification of the overall agreement between classifications by Bland-Altman test showed that: (i) AACE/ACE-AME is the least interchangeable with all the other three classification systems (Figures 4A,D,E), (ii) MUT is comparable to both ATA and BTA (Figures 4B,C), and (iii) ATA and BTA are highly interchangeable (Figure 4F).

Finally, the agreement between single nodule analyses by different classifications was evaluated considering Weighted Cohen's Kappa (Table 3). ATA and BTA had the best agreement (κ = 0.723). AACE/ACE-AME showed slight agreement with BTA (κ = 0.177) and MUT (κ = 0.183), and fair agreement with ATA (κ = 0.282). Finally, MUT had fair agreement with both ATA (κ = 0.291) and BTA (κ = 0.271).

**Table 3:** Frequencies, κ-value, significance and reproducibility of the different measurement of classification systems are illustrated.

		**MUT**					**κ**	**Significance**
ATA		**1**	**2**	**3**	**4**	**5**	0.291	Fair agreement
	1	0	56	3	0	0		
	2	1	7	42	1	0		
	3	0	7	54	2	0		
	4	0	3	73	3	0		
	5	6	6	79	88	26		
BTA							0.271	Fair agreement
	1	0	0	0	0	0		
	2	1	67	55	2	0		
	3	1	1	23	2	0		
	4	0	3	54	1	0		
	5	5	8	119	89	26		
AACE/ACE/AME							0.183	Slight agreement
	1	0	57	24	1	0		
	2	1	16	77	2	0		
	3	6	6	150	91	26		
		**AACE/ACE-AME**						
ATA		**1**	**2**	**3**			0.282	Fair agreement
	1	59	0	0				
	2	16	34	1				
	3	7	51	5				
	4	0	3	76				
	5	0	8	197				
BTA							0.177	Slight agreement
	1	0	0	0				
	2	70	53	2				
	3	10	9	8				
	4	0	0	58				
	5	2	34	211				
		**ATA**						
BTA		**1**	**2**	**3**	**4**	**5**	0.723	Substantial agreement
	1	0	0	0	0	0		
	2	59	40	25	0	1		
	3	0	10	14	0	3		
	4	0	0	0	52	6		
	5	0	1	24	27	195		

*The interpretation of agreement is: less than chance (κ = 0), slight (κ = 0.01–0.20), fair (κ = 0.21–0.40), moderate (κ = 0.41–0.60), substantial (κ = 0.61–0.80), or almost perfect agreement (κ = 0.81–0.99)*.

*AACE/ACE-AME Task Force on thyroid nodules, American Thyroid Association (ATA), British Thyroid Association (BTA), and Modena US Thyroid Classification (MUT)*.

## Discussion

The results of the present study demonstrate that US operator subjective impression has a not negligible role in defining the risk of malignancy of a thyroid nodule. In fact, when a system like MUT is used, the highest accuracy is reached, overcoming ATA, BTA, and AACE/ACE-AME classifications. Moreover, MUT has high specificity, maintaining good sensitivity. This result suggests that the operator subjective impression, resulting from US technique knowledge and clinical experience, has a considerable impact on US accuracy. Thus, objective US findings need to be critically processed by the operator, who can re-weight the risk with respect to what is established by the classification system alone. Thus, in the clinical daily life thyroid US remains a subjective imaging tool that holds the operator expertise as an intrinsic characteristic of this instrument (24, 25).

From a practical point of view, nodules considered as suspect or very suspect (MUT class 4 or 5) by the US operator need to undergo FNA, since the malignancy risk is of 63 and 90%, respectively. Whereas cytological investigation must be considered in nodules classified as AACE/ACE-AME 3, ATA 5, or BTA5.

Then, we evaluated the interchangeability between classifications and the degree of agreement in the evaluation of each single nodule. MUT differs from ATA and BTA, which are highly exchangeable and mostly in agreement with each other. AACE/ACE-AME is the one that differs most from all the others, probably also as a consequence of its peculiar structure that provides only three classes instead of five. In conclusion, we think that a classification that considers the operator's subjectivity is inevitably different from the others, but in the end leads to better accuracy.

The main limit of guidelines proposed by international societies derives from the lack of specificity, mostly at highest categories. In fact, we confirm that they all have high sensitivity but low specificity (18, 21, 28). Accordingly, malignancy rate remains between 41 and 50% even in the highest risk class, with possible consequent over-medicalization and unnecessary FNA. In particular, our results show a much lower percentage than that expected for ATA classification. Guidelines and following studies indicate a malignancy rate above 70% for ATA high suspicion lesions (16, 21, 28). On the contrary, our results agree with recently published data, demonstrating a positive predictive value of 28% (29) and a malignancy rate of 55% (18) in ATA highest risk class. Even Lauria Pantano et al. (19) found a lower rate of malignancy within ATA high-suspicion (35.6%) and AACE/ACE-AME class 3 (19.8%) compared to what was expected. Finally, a considerable unnecessary FNA rate was demonstrated for the highest category of both ATA (52%) and AACE/ACE-AME (32%) (21).

It must be here emphasized that guideline classifications have been proposed as a careful and reasoned outcome of the scientific literature. However, the use of rigid rating systems is not always easy in real clinical life because of the nearly endless number of US feature combinations. Sometimes the clinician struggles to enter the lesion in a class rather than another, because of intermediate or ambiguous situations (14, 18, 19, 25). Moreover, interobserver agreement has recently been questioned in a multicenter study, resulting lower than that suggested by single center studies (24). Finally, it is possible that a nodule is classified in a category that does not match the one in which the clinician would have classified it according to his/her experienced-based perception.

We think that the present study has several strengths: all the US examinations were performed by the same expert endocrinologist, who prospectively evaluated patients with indeterminate or suspect FNA results; detailed and systematic collection of sonographic findings was compiled for each nodule, making the information available for a subsequent, retrospective, application of other classifications; the availability of histological diagnosis allowed to calculate the real diagnostic accuracy of each US risk class; in multinodular goiter, all the nodules, not only the ones addressed to FNA, were US evaluated and correlated with histological outcome, representing a good sample to test classifications performance.

However, some limitations must be listed. First of all, subjective impression is by definition difficult to quantify and classify. MUT is just an example, used as a tool to blow up the importance of subjectivity. Then, US performer was aware of the presence of a suspect nodule in the examined thyroid, which may have affected his judgment, especially in solitary nodules. Moreover, it is likely that our result is affected by a selection bias due to the fact that all the selected solitary nodules were cytologically indeterminate or suspect and already addressed to surgery. Lastly, the retrospective assignment to AACE/ACE-AAME, ATA, and BTA categories does not allow the evaluation of the operator compliance with their use nor the unavoidable inter-operator variability in US interpretation (30). The presence of a single operator ensures judgment homogeneity, but at the same time it avoids any inter-observer evaluation, making the use of MUT not generalizable. Further studies will be needed for this purpose.

In clinical practice, the present study suggests that the “human brain factor,” meaning the overall impression of the US operator, is of value to better stratify malignancy risk after having applied a US classification. Secondly, we confirm that the use of one of the available classifications must be encouraged since it helps to correctly address risky nodules to FNA (22). Since most of them are highly interchangeable, it does not matter which one is used, but the operator must be aware of its limits and compensate with his/her subjective impression. US operator should also keep in mind that US classifications are targeted to differentiated thyroid carcinoma and may fail in identifying the most worrisome subtypes of thyroid cancer, such as the anaplastic one, presenting different and misleading US-appearances (31). The results of this study suggest that the operator have to embrace one of the available US classifications in clinical practice, but he/she should bear in mind that these classifications do not cover the wide umbrella of heterogeneous characteristics of thyroid nodules since they operate by simplification within each US category and that inter- and intra-observer differences in the interpretation of US characteristics exist. Thus, the US operator may avoid to rigidly categorize the nodule according the rigid criteria of the classification especially when the characteristics of the nodule do not clearly fit with these criteria. By reconverting the US category according to the self-judgment of the operator, in fact, may improve the diagnostic accuracy of US, especially in case of operator with a great experience in thyroid US. However, the presence of a highly experienced US-physician is not guaranteed in low-volume thyroid centers, where the application of classification could represent a useful and effective first approach.

In conclusion, our findings suggest the good diagnostic performance of US classifications, corroborating the thesis in favor of their usefulness in clinical life. But accuracy improves when the subjective impression of the clinician is considered too. In this sense, the inescapable subjectivity of the sonographic description is no more a limit, but it could be an added value for the endocrinologist, who is able to integrate the guidelines derived ranking with his/her experienced-based perception.

## Data Availability Statement

The raw data supporting the conclusions of this article will be made available by the authors, without undue reservation.

## Ethics Statement

The studies involving human participants were reviewed and approved by Comitato etico provinciale di Modena. The patients/participants provided their written informed consent to participate in this study.

## Author Contributions

BM and VR contributed to the study conception and design. Material preparation, data collection were performed by BM, GB, AA, ET, MM, and analysis were performed by GB, SK, and BM. The first draft of the manuscript was written by GB, BM, VR, and MS. All authors commented on previous versions of the manuscript, read and approved the final manuscript.

## Conflict of Interest

The authors declare that the research was conducted in the absence of any commercial or financial relationships that could be construed as a potential conflict of interest.
